# Effects of a Behavioral Economics Intervention on Food Choice and Food Consumption in Middle-School and High-School Cafeterias

**DOI:** 10.5888/pcd15.170377

**Published:** 2018-07-05

**Authors:** Emilee L. Quinn, Donna B. Johnson, Mary Podrabsky, Brian E. Saelens, Wesley Bignell, James Krieger

**Affiliations:** 1 Center for Public Health Nutrition, Department of Health Services, University of Washington, Seattle, Washington; 2 Center for Public Health Nutrition, Department of Health Services, and Nutritional Sciences Program, University of Washington, Seattle, Washington; 3 Seattle Children’s Research Institute and Department of Pediatrics in the School of Medicine, University of Washington, Seattle, Washington; 4 Department of Sociology, University of Washington, Seattle, Washington; 5 Healthy Food America Seattle, Washington; Departments of Medicine and Health Services, University of Washington, Seattle, Washington

## Abstract

**Introduction:**

Changing food choice architecture in school cafeterias through behavioral economics may increase student selection and consumption of healthy foods. However, most research assesses the effects of short-term interventions. We evaluated a year-long choice architecture intervention implemented by school food service staff.

**Methods:**

Food service staff from 6 secondary schools in one school district received training and support to implement behavioral economics strategies in their cafeterias to promote student selection of fruit, vegetables, and low-fat white milk. We compared student selection and consumption of these foods in the intervention schools to 5 comparison schools in the same district on the basis of visual assessment of plate waste. We applied a difference-in-differences approach to estimate intervention effect.

**Results:**

Data for 902 students were assessed at baseline, and data for 1,407 were assessed at follow-up. In fully adjusted analyses for all students, there were significantly greater absolute increases in the proportions of intervention school students selecting any fruit, including (0.09) and excluding (0.16) juice, and students selected more fruit items including (0.21) and excluding (0.17) juice. The absolute increase in proportion of intervention students consuming fruit excluding juice (0.14) was significantly greater. However, in some analyses, fewer intervention students who selected fruits or vegetables ate them, or they ate fewer of them. There were no intervention effects for vegetables or low-fat white milk.

**Conclusion:**

Our results indicate that behavioral economics–based choice architecture can promote student selection of healthy foods, but they raise questions about whether it increases their consumption.

## Introduction

School meals reach more than 31 million US students daily, and more than 70% are served to low-income children (1). Nutritious school food can reduce health disparities and improve students’ diet and health (2–4). Changes in federal standards for the National School Lunch Program increased availability of healthy school food (5), although some of the food may go uneaten (6).

Choice architecture based on principles of behavioral economics is used to encourage children to choose healthy foods at school (7–10). Behavioral economics is rooted in psychology research, showing that subtle environmental factors can influence decisions and behaviors (11). In school lunchrooms, choice architecture consists of small, low-cost changes in convenience, attractiveness, and visibility of foods to encourage healthier choices. Such changes include offering healthy items as default selections, in “grab-and-go” combinations, with creative names, and in multiple locations (12).

Although choice architecture may improve students’ selection and consumption of healthy food (13–15), rigorous studies are few and inconclusive (16,17), and many use small sample sizes (18–20), lack control conditions (14,15,18,19), or last for short periods (13,18). Researchers, not school staff, designed and implemented many of the interventions, which limits replicability. We evaluated whether a year-long choice architecture intervention implemented by school cafeteria managers changed student selection and consumption of healthy foods.

## Methods

### Intervention

The Centers for Disease Control and Prevention’s Community Transformation Grants (CTG) funded projects that change environments and systems to support healthy living (www.cdc.gov/communitytransformation). The King County, Washington, CTG focused on changes across sectors in geographic areas with health inequities. In one project, a large, racially diverse, suburban school district applied for and received funding to support its food service staff in implementing choice architecture strategies (21).

**Figure 1:**
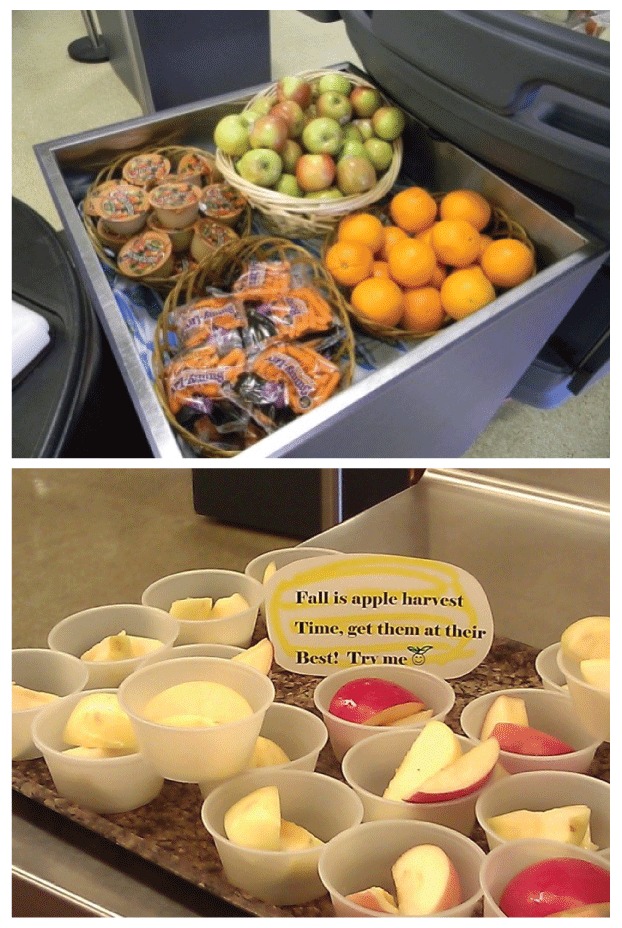
Displays from a behavioral economics intervention on the effects on food choices and food consumption in middle-school and high-school cafeterias, King County, Washington, 2013–2014.

Kitchen managers and staff from secondary schools selected for the intervention participated in an initial workshop in April 2013 that was facilitated by a nationally recognized expert on behavioral economics. The half-day training presented evidence-based behavioral economics principles for changing school lunchrooms (www.smarterlunchrooms.org). Strategies presented included displaying items in attractive containers, offering precut fruit, using creative names, using signage, placing items strategically (eg, at eye-level and/or in multiple places), and having staff prompt students (Figure 1).

At the beginning of the 2013–2014 school year, a technical team (district dietitian, kitchen manager “leader,” university-based school nutrition specialist, and CTG project lead) assisted the kitchen managers in developing work plans based on strategies covered in the training. The team provided implementation support throughout the school year through site visits and telephone calls (one per school per month, on average). At a second half-day workshop for participants in October 2013, the content expert reviewed principles and strategies and facilitated discussion of successes and challenges. The project budget included up to $2,000 per school for promotional materials and supplies such as fruit bowls; several schools purchased larger equipment (eg, milk coolers, salad bars).

### Study design and sampling strategy

For this quasi-experimental study, we compared student selection and consumption of fruit (eg, 100% orange or apple juice, apples, grapes, applesauce), vegetables (eg, roasted or par-fried potatoes, side salads, carrots), and low-fat white milk (LFW) at the beginning and end of the school year in 6 intervention and 5 comparison schools from the same district.

Based on CTG’s health equity goals, the district’s nutrition services department selected the secondary schools in the district with the highest rates of free and reduced-price lunch eligibility (FRPE) to participate in the intervention (3 middle and 3 high schools). The district’s remaining 3 middle and 2 high schools comprised the comparison group.

Data were collected during one visit to each school cafeteria (all lunch periods) in September and October of 2013 at the beginning of the intervention and late in the intervention in May 2014 (eg, 2 total visits for each school). Kitchen managers selected visit dates from several offered by the data collection team. Lunchroom tables were numbered and selected using a random number generator. As students sat at selected tables with lunch trays, trained data collectors asked for students’ verbal consent to observe their lunch selections (one tray per student). Food items not part of the school meal were not evaluated. Data collectors observed as many trays as time allowed using a predetermined recruitment path based on table set-up. The data collection team was larger at follow-up, resulting in a larger sample size.

### Data collection

Using validated and reliable methods (22,23), data collectors observed the type and quantity of items selected by each student, noted this on a data collection card, and taped the card to the student’s tray. The data collector asked the student to take their tray, with all remaining packaging and uneaten food, to a labeled rack once finished eating. There, data collectors removed the cards from trays and estimated the remaining proportion of each food item in 25% increments using displayed reference portions. Serving sizes were standard for each item type. Made-to-order entrée salads were excluded from analysis since they were only offered at follow-up. The University of Washington institutional review board approved the study protocol.

### Data analysis

We addressed 5 categories of targeted healthy food and beverages: fruit including and excluding juice; vegetables including and excluding potatoes; and LFW milk. For each student, the numbers of healthy items selected (eg, juice carton, bag of grapes) in each category were summed to create one “items selected” variable per category. Likewise, we created 5 “amount consumed” variables in each category by subtracting the sum of all proportions of unconsumed selected items from the total number of items selected for each student.

Aggregating these student-level variables, we calculated 1) the proportion of students who selected any fruit, vegetable, or LFW milk; 2) the proportion of students who consumed any, defined as 25% or more fruit, vegetable, or LFW milk; 3) the average number of fruit, vegetable, or LFW milk items selected per student; and 4) the average number or amount of fruit, vegetable, or LFW milk items that students consumed. Items recorded as selected but for which no expected evidence remained (ie, milk carton) were included in selection but not consumption analyses. We examined consumption among the full sample and subsamples of students who selected items (eg, for fruit consumption, entire student sample, and the sample of students who selected fruit).

We applied a difference-in-difference approach to measure the average intervention effect. This approach assessed how the intervention schools performed relative to the comparison schools by comparing the change experienced by each of the 2 groups from baseline to follow-up. We used bivariate analyses (*t* tests and tests of differences in proportions) to assess differences between groups at baseline and follow-up for each variable, and differences in means and proportions between points in each group. We performed ordinary least squares regression analyses to obtain simple difference-in-difference estimates and standard errors for each continuous variable. We also used linear probability models for binary dependent variables, indicating whether a student selected or consumed any of the particular food items. We conducted regression analyses using models that adjusted for school-level covariates (ie, middle school versus high school, proportion of FPRE students, and proportion of white students). Data were analyzed using Stata version 13 (StataCorp LP). Significance was set at *P* < .05 for all tests.

## Results

**Figure 2:**
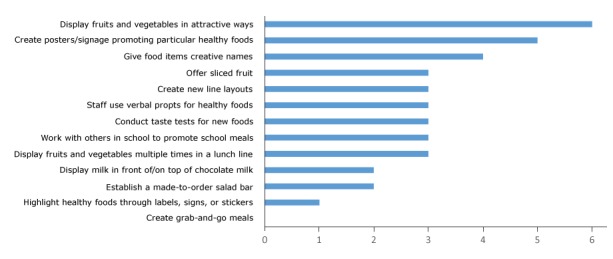
Number of kitchen managers (N = 6) in intervention schools who tried 13 behavioral economics strategies, intervention on effects on food choices and food consumption in middle-school and high-school cafeterias, King County, Washington, 2013–2014. **Table:** 

Intervention schools had greater proportions of student FRPE (by d­­esign) and African American and Hispanic students (Table 1). On the basis of being perceived by kitchen managers as most feasible and effective in promoting student selection, displaying fruits and vegetables in attractive ways and using signage to promote healthy foods sliced fruit were the strategies most often tried (Figure 2). All intervention schools reported displaying fruit and vegetables in attractive ways; 2 reported making LFW milk more visible than chocolate, and none reported creating “grab and go” meals.

**Table 1:** Characteristics of Schools Enrolled in Cafeteria Intervention Study, by Intervention and Comparison Group, King County, Washington, 2013–2014

Characteristic	Study Schools^a^		
	Intervention Group (n = 6)	Comparison Group (n = 5)	*P* Value
**Average number of students enrolled in each school^a^**	1,026	1,219	.68
**Average percentage of students eligible for FRPE^b^**	58.3	35.3	.005
**Average percentage of student body, by race^a^**			
American Indian/Alaska Native	0.6	0.7	.23
Asian	17.8	15.8	.68
Black/African American	14.6	9.2	.04
Hawaiian/other Pacific Islander	3.1	1.7	.06
White	33.6	50.9	.06
Two or more races	7.3	8.6	.24
**Average percentage of student body by ethnicity, % Hispanic^a^**	23.0	13.1	.01

Abbreviation: FRPE, Free and Reduced Price Lunch eligibility.

a Data from Washington State Office of Superintendent of Public Instruction, December 2013 Enrollment Reports.

b Data from the 2013–2014 Washington Public School Free and Reduced Price Meal Eligibility report for October 31, 2013.

Behavioral Economics Strategy	No. of Managers Who Tried Strategy
Create grab and go meals	0
Highlight healthy foods through labels, signs, or stickers	1
Establish a made-to-order salad bar	2
Display milk in front of/on top of chocolate milk	2
Display fruits and vegetables multiple times in a lunch line	3
Work with others in school to promote school meals	3
Conduct taste tests for new foods	3
Staff use verbal prompts for healthy foods	3
Create new line layouts	3
Offer sliced fruit	3
Give food items creative names	4
Create posters/signage promoting particular healthy foods	5
Display fruits and vegetables in attractive ways	6

Nearly all students (93%) invited to participate in the study consented to do so; students returned 88% of trays with cards attached for assessment. Data for 2,309 trays were included in analyses. At baseline, 416 intervention and 486 comparison trays were assessed. At follow-up, 734 were assessed for the intervention group, and 673 for the comparison group. The comparison group sample had a smaller proportion of assessed trays belonging to female students at follow-up (Table 2).

**Table 2:** Differences in Participants Enrolled in Cafeteria Intervention Study, by Baseline Group and Follow-Up Group, King County, Washington, 2013–2014

Characteristic	Study Participants				Baseline Difference, *P* Value^a^	Follow-Up Difference, *P* Value^a^
	Baseline (n = 416), No. (%)	Follow-Up (n = 734), No. (%)	Baseline (n = 486), No. (%)	Follow-Up (n = 673), No. (%)		
**School level**						
Middle school	239 (57)	394 (54)	265 (55)	378 (56)	.38	.35
High school	177 (43)	340 (46)	221 (45)	295 (44)		
**Sex**						
Female	161 (39)	335 (46)	205 (42)	245 (36)	.52	<.001
Male	223 (54)	360 (49)	249 (51)	368 (55)		
Unrecorded	32 (8)	39 (5)	32 (7)	60 (9)		

a Differences in frequencies between intervention and comparison groups at baseline and follow-up were assessed using χ^2^ *t* tests.

### Proportion of students selecting items

At baseline, selection of foods was similar between intervention and comparison groups, except for the proportion of students selecting vegetables including potatoes, which was higher for the comparison group (Table 3). In both groups, fruit including juice was the only category for which more than half of all students selected an item. Less than one-quarter of students selected LFW milk or vegetables excluding potatoes.

**Table 3:** Differences in Proportion of Students Selecting and Consuming Foods, Cafeteria Intervention Study, King County, Washington, 2013–2014

Food Category	Within Groups^a^						Between Groups^a^^,^ b				
	Intervention Group			Comparison Group							
	Proportion (No.) at Baseline	Between Baseline and Follow-Up		Proportion (No.) at Baseline	Between Baseline and Follow-Up		Baseline Diff, *P*	Between Baseline and Follow-Up (Diff-in-Diff)			
		Diff	*P*		Diff	*P*		Unadj	*P*	Adj^c^	*P*
**Students’ selecting any**											
Fruit, including juice	0.84 (416)	0.04	.09	0.88 (486)	−0.05	.02	.07	0.09	.004	0.09	.004
Fruit, excluding juice	0.30 (416)	0.12	<.001	0.33 (486)	−0.05	.06	.39	0.17	<.001	0.16	<.001
Vegetables, including potatoes^d^	0.38 (416)	0.28	<.001	0.49 (486)	0.24	<.001	.001	0.04	.30	0.03	.30
Vegetables, excluding potatoes	0.19 (416)	0.02	.39	0.20 (486)	−0.05	.03	.69	0.07	.04	0.05	.11
Low-fat white milk	0.11 (416)	0.04	.06	0.15 (486)	0.02	.42	.11	0.02	.47	0.02	.55
**Students consuming any^e^**											
Fruit, including juice	0.79 (371)	0.02	.35	0.80 (435)	−0.02	.45	.79	0.04	.23	0.04	.24
Fruit, excluding juice	0.19 (393)	0.12	<.001	0.23 (457)	−0.02	.40	.17	0.14	<.001	0.14	<.001
Vegetables, including potatoes^d^	0.34 (416)	0.28	<.001	0.42 (486)	0.27	<.001	.01	0.01	.81	0.00	.92
Vegetables, excluding potatoes	0.14 (416)	0.05	.05	0.14 (486)	−0.01	.62	.78	0.06	.07	0.04	.20
Low-fat white milk	0.09 (411)	0.04	.03	0.12 (478)	0.02	.23	.10	0.02	.56	0.01	.66
**Students consuming any (of those who selected)^f^**											
Fruit, including juice^d^	0.96 (305)	−0.02	.18	0.92 (378)	0.03	.05	.02	−0.05	.01	−0.05	.02
Fruit, excluding juice	0.74 (102)	0.11	.02	0.81 (130)	0.11	.01	.19	−0.01	.93	−0.01	.89
Vegetables, including potatoes	0.90 (158)	0.04	.06	0.87 (236)	0.09	<.001	.43	−0.04	.16	−0.05	.13
Vegetables, excluding potatoes	0.76 (79)	0.14	.003	0.68 (98)	0.15	.01	.27	−0.01	.92	0.03	.70
Low-fat white milk	0.85 (41)	0.03	.61	0.90 (63)	0.07	.03	.43	−0.04	.52	−0.04	.61

Abbreviations: adj, adjusted; diff, difference; unadj, unadjusted.

a Differences in proportions between group and time periods were assessed using proportions *t* tests. Positive values favor intervention group, and negative values favor the comparison group.

b Difference-in-difference estimates were assessed using ordinary least squared regression.

c Results of models adjusted for school-level covariates (Free and Reduced Price Lunch eligibility, race, and school level).

d Significant differences between groups existed at baseline.

e The denominator for “students consuming” percentages vary for each cell and reflect cases with nonassessable data (eg, no juice cup on tray).

f The denominator for “students consumed (of those who selected)” varies for each cell and reflects only cases that selected items from that category with assessable data.

The proportion of students who selected fruit excluding juice and vegetables including potatoes increased significantly in intervention schools from baseline to follow-up (Table 3). Patterns were mixed for the comparison group, with a significant increase in the proportion of students selecting vegetables that included potatoes, but decreases in the proportion of students selecting fruit including juice and vegetables excluding potatoes.

The change in proportions of students selecting the items from baseline to follow-up was significantly greater for the intervention group for fruit including juice, fruit excluding juice, and vegetables excluding potatoes, though the difference-in-difference for vegetables excluding potatoes did not remain significant in adjusted analyses.

### Proportion of students consuming items

At baseline, consumption of foods was similar between intervention and comparison groups, except for the overall proportion of students consuming vegetables including potatoes, which was higher for comparison group students, and the proportion of students selecting fruit including juice and consuming it, which was higher for the intervention group (Table 3). In both groups, fruit including juice was the only category for which more than half of all students consumed an item. Consumption among students selecting items was high. Less than one-quarter of students consumed any LFW milk or vegetables excluding potatoes.

The proportions of intervention school students consuming any of the items increased significantly for fruit excluding juice and vegetables including potatoes, and LFW milk. For the comparison group, the only significant change was an increase in the proportions of students consuming vegetables including potatoes. Among students selecting the items, the proportion of intervention school students consuming fruit excluding juice and vegetables excluding potatoes increased significantly; proportions of comparison group students consuming foods increased significantly across all categories.

Across all students, the change in proportions of intervention school students from baseline to follow-up consuming fruit excluding juice was significantly higher than that of comparison school students. When restricted to students who selected the items, the comparison group had significantly increased consumption of fruit including juice.

### Quantity of items selected by students

The average numbers of items selected by students at baseline were not significantly different between intervention and comparison groups, with the exception of vegetables including potatoes, which was higher in comparison schools (Table 4). Students in both groups selected the greatest quantity of items from the fruit including juice category.

**Table 4:** Differences in Mean Number of Foods Selected and Consumed by Students, Cafeteria Intervention Study, King County, Washington, 2013–2014

Food Category	Within Groups^a^						Between Groups^a^^,^ b				
	Intervention			Comparison							
	Mean No. (n), Baseline	Between Baseline and Follow-up		Mean No. (n), Baseline	Between Baseline and Follow-up		Baseline Diff, *P*	Unadj	Between Baseline and Follow-up (Diff-in-Diff)		
		Diff	*P*		Diff	*P*			*P*	Adj^c^	*P*
**Items selected**											
Fruit, including juice	1.13 (416)	0.16	<.001	1.16 (486)	−0.05	.21	.40	0.22	.001	0.21	.001
Fruit, excluding juice	0.32 (416)	0.15	<.001	0.35 (486)	−0.03	.32	.39	0.18	<.001	0.17	<.001
Vegetables, including potatoes^d^	0.46 (416)	0.32	<.001	0.58 (486)	0.29	<.001	.006	0.03	.60	0.01	.89
Vegetables, excluding potatoes	0.21 (416)	0.01	.60	0.22 (486)	−0.05	.05	.88	0.06	.09	0.04	.24
Low-fat white milk	0.11 (416)	0.04	.07	0.15 (486)	0.01	.51	.11	0.02	.45	0.02	.51
**Items consumed^e^**											
Fruit, including juice	0.94 (371)	0.08	.08	0.95 (435)	0.01	.84	.76	0.07	.25	0.07	.30
Fruit, excluding juice	0.17 (393)	0.11	<.001	0.19 (457)	0.03	.31	.45	0.08	.04	0.08	.05
Vegetables, including potatoes^d^	0.35 (416)	0.23	<.001	0.43 (486)	0.27	.00	.04	−0.04	.39	−0.06	.21
Vegetables, excluding potatoes	0.13 (416)	0.02	.48	0.11 (486)	0.00	.79	.47	0.02	.48	0.01	.83
Low-fat white milk	0.08 (411)	0.03	.07	0.11 (478)	0.02	.27	.13	0.01	.65	0.01	.72
**Items consumed (of those who selected)^f^**											
Fruit, including juice	1.14 (305)	0.04	.41	1.09 (378)	0.08	.06	.30	−0.04	.53	−0.05	.45
Fruit, excluding juice	0.64 (102)	0.11	.09	0.65 (130)	0.29	<.001	.82	−0.18	.04	−0.19	.03
Vegetables, including potatoes	0.92 (158)	−0.03	.45	0.88 (236)	0.10	.01	.43	−0.13	.03	−0.14	.02
Vegetables, excluding potatoes	0.69 (79)	0.00	.96	0.57 (98)	0.15	.03	.14	−0.14	.13	−0.12	.19
Low-fat white milk	0.77 (41)	0.00	.96	0.80 (63)	0.06	.25	.67	−0.06	.46	−0.05	.60

Abbreviations: adj, adjusted; diff, difference; prop, proportion; unadj, unadjusted.

a Differences in means between group and time periods were assessed using proportions *t* tests. Positive values favor intervention group, and negative values favor the comparison group.

b Difference-in-differences estimates were assessed using ordinary least squared regression.

c Results of models adjusted for school-level covariates (Free and Reduced Price Lunch eligibility, race, and school level).

d Significant differences between groups existed at baseline.

e Cases for “items consumed” averages excluded cases with nonassessable data (eg, no juice cup on tray).

f Cases for “items consumed (of those who selected)” include cases which selected items from that category with assessable data.

Between baseline and follow-up, students in the intervention schools selected significantly more items in 3 of the 5 categories: fruit including juice, fruit excluding juice, and vegetables including potatoes. For the comparison group, only the change in number of vegetables including potatoes selected increased significantly.

The increase in the mean number of items selected by students was significantly greater for the intervention than the comparison group for fruit including and excluding juice.

### Quantity of items consumed by students

The average numbers of items consumed by students at baseline were not significantly different for intervention versus comparison groups with the exception of vegetables including potatoes, which was higher in comparison schools (Table 4). Students in both groups consumed the greatest quantity of items from the fruit including juice category.

The quantity of items consumed by intervention students increased significantly for fruit excluding juice and vegetables including potatoes. Changes in the average number of items consumed by students in the comparison group were significant only for vegetables including potatoes. When restricted to those who selected the items, the proportion of intervention students eating items in any category did not change; the proportion of comparison group students consuming fruit excluding juice and vegetables including and excluding potatoes increased significantly.

Intervention group students had a greater increase than comparison group students in the amount of fruit excluding juice eaten, but the difference-in-difference did not remain significant in adjusted analyses. When restricted to students who selected the items, students in the comparison group had a significantly greater change in average consumption of fruit excluding juice and vegetables including potatoes than intervention school students.

## Discussion

We measured treatment effects of a year-long behavioral economics intervention implemented by kitchen managers in secondary school cafeterias. The intervention encouraged more students (eg, a greater proportion of students) to choose fruit and fruit juice and encouraged those students to take a greater quantity of fruit and juice items, but to have limited effects on healthy food consumption. Estimates suggest that 9% and 16% more students exposed to choice architecture selected fruit (including and excluding juice, respectively) and more fruit items compared with comparison schools. Fruit consumption effects were more limited. Although 12% more students in the intervention group consumed some fruit (excluding juice) compared with the −2% change in the comparison schools, they did not consume a greater amount on average. In some analyses restricted to students who had selected specific items, fewer students exposed to the intervention ate those fruit or vegetables, or they ate less of them. There were no other changes in selection or consumption of vegetables or LFW milk attributable to the intervention. From an overall student population perspective, while more intervention students consumed fruit excluding juice, there was no significant increase in the amounts of healthy items consumed.

These mixed results align somewhat with those of other studies. One short-term study of behavioral economics in 2 secondary schools without a comparison group demonstrated similar results in increased selection and consumption of fruit but also greater increases in selection and consumption of nonstarchy vegetables (19). Another longer-term randomized clinical trial found that choice architecture alone increased selection but not consumption of fruit or vegetables (17). Several studies indicate that encouraging students to voluntarily take and drink unflavored milk is often challenging (17,24). Other studies also indicate that fruit may be more easily promoted than vegetables among children (16,25,26).

That consumption improved more among comparison group students in some cases is worth further exploration. Our results indicate that choice architecture may increase healthy choices but that such choices do not necessarily lead to consumption (17). Taste preferences may play a greater role, and preferred foods like fruit may be more likely to be eaten once selected. Research shows that offering a greater variety of fruit and vegetables (16,26) and improving taste or quality (17) can increase consumption. More research is needed to develop interventions that affect consumption once a food is chosen.

Interest in behavioral economics to enhance child nutrition programs has increased with the backing of the US Department of Agriculture, which administers federal school meal programs (8,10,27). Many US schools now use these strategies (28). Despite this interest and compelling data on particular strategies (13,15,16,18–20,29), little is known about the best way to achieve broad, sustainable, and effective implementation of choice architecture in school cafeterias. Additional evaluations are needed to assess how school lunch professionals translate research knowledge into practice, as well as particular strategies. For example, kitchen managers may find strategies easier to apply to fruit and vegetables than to LFW milk as evidenced by the strategies tried in this study. Also, certain strategies may influence student selection and consumption differently.

Our study’s strengths include its comparison group design, large sample size, year-long duration, implementation by food service staff, and reliance on objective observational data for selection and consumption. Our study also has limitations. Schools were not randomly assigned to groups, nor were they equivalent at baseline, though use of adjusted difference-in-difference analyses helped address these shortcomings. Managers from the comparison group may have heard about and tried intervention strategies. Although lunch menus varied across days of data collection and seasonality may have influenced results, the types of fruits, vegetables, and milk offered were similar across all schools and at both time points. As with other plate waste studies (6), some data were lost when students threw away their food or packaging or took it out of the cafeteria.

School nutrition services aim to operate fiscally solvent, healthy, and appealing meal programs. These findings can help school nutrition and public health stakeholders understand how subtle changes in cafeteria environments made by food service staff influence students. Our findings add to the evidence that behavioral economics–based choice architecture may promote student selection of some healthy foods in a readily implemented way, but they also raise questions about whether it can increase consumption of these foods.

## References

[1] Food Research and Action Center. National School Lunch Program; 2016. http://frac.org/wp-content/uploads/cnnslp.pdf. Accessed May 23, 2017.

[2] Centers for Disease Control and Prevention (CDC). School health guidelines to promote healthy eating and physical activity. MMWR Recomm Rep 2011;60(RR–5, No. RR-5):1–76. 21918496

[3] Terry-McElrath YM , O’Malley PM , Johnston LD . Foods and beverages offered in US public secondary schools through the National School Lunch Program from 2011–2013: early evidence of improved nutrition and reduced disparities. Prev Med 2015;78:52–8. 10.1016/j.ypmed.2015.07.010 26190369

[4] Terry-McElrath YM , O’Malley PM , Johnston LD . Potential impact of national school nutritional environment policies: cross-sectional associations with US secondary student overweight/obesity, 2008-2012. JAMA Pediatr 2015;169(1):78–85. 10.1001/jamapediatrics.2014.2048 25402551

[5] Healthy, Hunger-Free Kids Act of 2010, Pub. L. No. 111–296 (December 13, 2010).

[6] Gase LN , McCarthy WJ , Robles B , Kuo T . Student receptivity to new school meal offerings: assessing fruit and vegetable waste among middle school students in the Los Angeles Unified School District. Prev Med 2014;67(Suppl 1):S28–33. 10.1016/j.ypmed.2014.04.013 24747044 PMC4199919

[7] Guthrie J , Newman C . Eating better at school: can new policies improve children’s food choices? United States Department of Agriculture Economic Research Service; 2013. http://www.ers.usda.gov/amber-waves/2013-september/eating-better-at-school-can-new-policies-improve-children%E2%80%99s-food-choices.aspx#.VDwLVbDF8uc. Accessed May 23, 2017.

[8] Behavioral economics. United States Department of Agriculture Economic Research Service; 2014. http://www.ers.usda.gov/topics/food-choices-health/food-consumption-demand/behavioral-economics.aspx#.VD2iErDF8uc. Accessed May 23, 2017.

[9] Mancino L , Guthrie JF . When nudging in the lunch line might be a good thing. Amber Waves 2009;7(1):32–8.

[10] HealthierUS School Challenge. Smarter lunchrooms resources. United States Department of Agriculture; 2016. http://healthymeals.nal.usda.gov/healthierus-school-challenge-resources/smarter-lunchrooms. Accessed May 23, 2017.

[11] Camerer C . Behavioral economics: reunifying psychology and economics. Proc Natl Acad Sci USA 1999;96(19):10575–7. 10.1073/pnas.96.19.10575 10485865 PMC33745

[12] Cornell Center for Behavioral Economics in Child Nutrition Programs. Smarter lunchrooms self-assessment scorecard; 2014. http://smarterlunchrooms.org/sites/default/files/lunchroom_self-assessmt_score_card.final_.4-3-14.pdf. Accessed October 13, 2014.

[13] Wansink B , Just D . Healthy foods first: students take the first lunchroom food 11% more often than the third. J Nutr Educ Behav 2011;43(4):S8. 10.1016/j.jneb.2011.03.032

[14] Wansink B , Just D , Smith L . Move the fruit: putting fruit in new bowls and new places doubles lunchroom sales. J Nutr Educ Behav 2011;43(4):S1.

[15] Wansink B , Just DR , Hanks AS , Smith LE . Pre-sliced fruit in school cafeterias: children’s selection and intake. Am J Prev Med 2013;44(5):477–80. 10.1016/j.amepre.2013.02.003 23597811

[16] Nørnberg TR , Houlby L , Skov LR , Peréz-Cueto FJ . Choice architecture interventions for increased vegetable intake and behaviour change in a school setting: a systematic review. Perspect Public Health 2016;136(3):132–42. 10.1177/1757913915596017 26265733

[17] Cohen JF , Richardson SA , Cluggish SA , Parker E , Catalano PJ , Rimm EB . Effects of choice architecture and chef-enhanced meals on the selection and consumption of healthier school foods: a randomized clinical trial. JAMA Pediatr 2015;169(5):431–7. 10.1001/jamapediatrics.2014.3805 25798990 PMC4540052

[18] Hanks AS , Just DR , Wansink B . Preordering school lunch encourages better food choices by children. JAMA Pediatr 2013;167(7):673–4. 10.1001/jamapediatrics.2013.82 23645188

[19] Hanks AS , Just DR , Wansink B . Smarter lunchrooms can address new school lunchroom guidelines and childhood obesity. J Pediatr 2013;162(4):867–9. 10.1016/j.jpeds.2012.12.031 23434267

[20] Hanks AS , Just DR , Smith LE , Wansink B . Healthy convenience: nudging students toward healthier choices in the lunchroom. J Public Health (Oxf) 2012;34(3):370–6. 10.1093/pubmed/fds003 22294661

[21] Cornell Center for Behavioral Economics in Child Nutrition Programs. 6 Guiding principles that improve school lunch eating behaviors; 2012. http://smarterlunchrooms.org/sites/default/files/introduction_to_smarter_lunchrooms_6_principles_powerpoint.pdf. Accessed October 13, 2014.

[22] Getts KM , Quinn EL , Johnson DB , Otten JJ . Validity and inter-rater reliability of the visual quarter-waste method for assessing food waste in middle and high school cafeteria settings. J Acad Nutr Diet 2017;117(11):1816–21. 10.1016/j.jand.2017.05.004 28688883 PMC7261231

[23] Hanks AS , Wansink B , Just DR . Reliability and accuracy of real-time visualization techniques for measuring school cafeteria tray waste: validating the quarter-waste method. J Acad Nutr Diet 2014;114(3):470–4. 10.1016/j.jand.2013.08.013 24135053

[24] Hanks AS , Just DR , Wansink B . Chocolate milk consequences: a pilot study evaluating the consequences of banning chocolate milk in school cafeterias. PLoS One 2014;9(4):e91022. 10.1371/journal.pone.0091022 24740451 PMC3989166

[25] Kim SA , Moore LV , Galuska D , Wright AP , Harris D , Grummer-Strawn LM , ; Division of Nutrition, Physical Activity, and Obesity, National Center for Chronic Disease Prevention and Health Promotion, CDC. Vital signs: fruit and vegetable intake among children — United States, 2003–2010. MMWR Morb Mortal Wkly Rep 2014;63(31):671–6. 25102415 PMC4584658

[26] Schwartz MB , Henderson KE , Read M , Danna N , Ickovics JR . New school meal regulations increase fruit consumption and do not increase total plate waste. Child Obes 2015;11(3):242–7. 10.1089/chi.2015.0019 25734372 PMC4484709

[27] United States Department of Agriculture. HealthierUS School Challenge: smarter lunchrooms. http://healthymeals.nal.usda.gov/hsmrs/2014HUSSCpromo/. Accessed May 23, 2017.

[28] Demissie Z , Brener ND , McManus T , Shanklin SL , Hawkins J , Kann L . School health profiles 2014: characteristics of health programs among secondary schools. Atlanta (GA): US Department of Health and Human Services, Centers for Disease Control and Prevention; 2015.

[29] Just DR , Wansink B . School lunch debit card payment systems are associated with lower nutrition and higher calories. Obesity (Silver Spring) 2014;22(1):24–6. 10.1002/oby.20591 23929600

